# Construction and validation of a 6-gene nomogram discriminating lung metastasis risk of breast cancer patients

**DOI:** 10.1371/journal.pone.0244693

**Published:** 2020-12-30

**Authors:** Lingchen Wang, Wenhua Wang, Shaopeng Zeng, Huilie Zheng, Quqin Lu

**Affiliations:** 1 Jiangxi Provincial Key Laboratory of Preventive Medicine, Nanchang University, Nanchang, Jiangxi, China; 2 Department of Biostatistics, School of Public Health, Nanchang University, Nanchang, Jiangxi, China; 3 Center for Experimental Medicine, The First Affiliated Hospital of Nanchang University, Nanchang, Jiangxi, China; Gangnam Severance Hospital, Yonsei University College of Medicine, REPUBLIC OF KOREA

## Abstract

Breast cancer is the most common malignant disease in women. Metastasis is the foremost cause of death. Breast tumor cells have a proclivity to metastasize to specific organs. The lung is one of the most common sites of breast cancer metastasis. Therefore, we aimed to build a useful and convenient prediction tool based on several genes that may affect lung metastasis-free survival (LMFS). We preliminarily identified 319 genes associated with lung metastasis in the training set GSE5327 (n = 58). Enrichment analysis of GO functions and KEGG pathways was conducted based on these genes. The best genes for modeling were selected using a robust likelihood-based survival modeling approach: GOLGB1, TMEM158, CXCL8, MCM5, HIF1AN, and TSPAN31. A prognostic nomogram for predicting lung metastasis in breast cancer was developed based on these six genes. The effectiveness of the nomogram was evaluated in the training set GSE5327 and the validation set GSE2603. Both the internal validation and the external validation manifested the effectiveness of our 6-gene prognostic nomogram in predicting the lung metastasis risk of breast cancer patients. On the other hand, in the validation set GSE2603, we found that neither the six genes in the nomogram nor the risk predicted by the nomogram were associated with bone metastasis of breast cancer, preliminarily suggesting that these genes and nomogram were specifically associated with lung metastasis of breast cancer. What’s more, five genes in the nomogram were significantly differentially expressed between breast cancer and normal breast tissues in the TIMER database. In conclusion, we constructed a new and convenient prediction model based on 6 genes that showed practical value in predicting the lung metastasis risk for clinical breast cancer patients. In addition, some of these genes could be treated as potential metastasis biomarkers for antimetastatic therapy in breast cancer. The evolution of this nomogram will provide a good reference for the prediction of tumor metastasis to other specific organs.

## 1. Introduction

Breast cancer is the most common malignant disease in women. In 2018, 2.1 million new cases of breast cancer were diagnosed among women worldwide, accounting for nearly a quarter of all female cancer cases [1]. However, with the progress of diagnosis and treatment technology, primary breast cancer does not pose a serious threat to patients’ lives; instead, metastasis is the foremost cause of death [2]. The 5-year survival rate for primary breast cancer patients is 99%, but it drops significantly to 26% when metastasis occurs [3]. The only way to help reduce the death rate of breast cancer patients is to effectively control and block tumor metastasis. More importantly, it has been noted that breast cancer has a proclivity to metastasize to specific organs, such as the lungs and bones [4]. The lung is one of the most common sites of breast cancer metastasis which can make a patient’s prognosis worse [5].

Metastasis of breast cancer, like other malignant tumors, is a complex biological process in which multiple genes interact and influence each other [6]. Tumor cells are regulated by a range of genes, including genes that promote metastasis and inhibit metastasis. Differences in the expression of these genes between patients determine the potential for and sites of tumor cell metastasis [7–9]. Screening of genes related to tumor metastasis can provide clues for studying tumor metastasis targets and predicting tumor metastasis sites [10, 11].

Significant developments in high-throughput techniques for genome-wide expression analysis and publicly available datasets have enabled us to analyze worldwide data [12]. Potential biomarkers and signaling pathways related to tumor cell metastasis could be screened using bioinformatics methods.

Previously, few studies have focused on the prediction of breast cancer metastasis to specific sites. In our study, we aimed to generate a useful and convenient prediction tool based on several genes that may affect lung metastasis-free survival (LMFS). Using one training dataset from the Gene Expression Omnibus (GEO), we identified 319 genes that were associated with lung metastasis in breast cancer. Six of these genes were further chosen using a robust likelihood-based survival modeling approach to build a gene prognostic nomogram. In addition, we tested the effectiveness of the nomogram in an independent validation set, manifesting its practical value for predicting the lung metastasis risk for clinical breast cancer patients. On the other hand, we found that neither the six genes in the nomogram nor the risk predicted by the nomogram were associated with bone metastasis of breast cancer, preliminarily suggesting that these genes and nomogram were specifically associated with lung metastasis of breast cancer. What’s more, five genes in the nomogram were significantly differentially expressed between breast cancer and normal breast tissues in the TIMER database. These genes could represent potential target genes for the treatment of metastatic breast cancer.

## 2. Material & methods

### 2.1 Microarray datasets from the gene expression Omnibus

We conducted a comprehensive search of breast cancer microarray datasets including lung metastasis information in the GEO database from the National Center for Biotechnology Information (NCBI) (http://www.ncbi.nlm.nih.gov/geo/). Only datasets with a sample size greater than 20 were selected for subsequent analysis. Then, the raw intensity files (CEL) of the datasets meeting our criteria for further analyses were downloaded from the GEO database. The robust multiarray average method of the R package “affy” was used to process raw intensity files and generate the gene expression matrices for each selected dataset [13]. The gene expression data of each sample were matched with the clinical information.

### 2.2 Univariate survival analysis

Log-rank tests for the high and low expression groups of each gene were performed using the R package “survival”. Lung metastasis of breast cancer was considered the outcome event. Genes with a P-value less than 0.01 were deemed candidate genes associated with lung metastasis in breast cancer for modeling.

### 2.3 Enrichment analysis of GO functions and KEGG pathways

Gene Ontology (GO) function and Kyoto Encyclopedia of Genes and Genomes (KEGG) pathway enrichment analyses were conducted using the WEB-based GEne SeT AnaLysis Toolkit (http://bioinfo.vanderbilt.edu/webgestalt/login.php) to understand the critical biological significance of the identified genes related to lung metastasis in breast cancer.

### 2.4 Selection of the best genes for constructing a gene prognostic nomogram

Among the genes related to lung metastasis in breast cancer, a robust likelihood-based survival approach was applied to select the best genes for building a gene prognostic nomogram. The whole selection process was implemented with the R package “rbsurv”. Details of the algorithm are summarized as follows:

1. All samples were randomly divided into the training set with N*(1 − p) samples and the validation set with N*p samples (p = 1/3). Then, the Cox proportional hazards model was used to fit a gene to the training set of samples to obtain the parameter estimate for this gene. Log-likelihood was evaluated with the parameter estimate and the validation set of samples. This process was implemented for each gene.
2. The above procedure was repeated 10 times; thus, 10 log-likelihoods were obtained for each gene. Next, the best gene g_(1)_ with the largest mean log-likelihood was selected. All the best lung metastasis survival-related genes were chosen by the robust likelihood-based method.
3. Let g_(1)_ be the chosen best gene in the previous step. Adjusting for g_(1)_, the second best gene was identified by repeating the above two steps. In other words, g_(1)_ + g_(j)_ was evaluated for every j, and an optimal two-gene model, g_(1)_ + g_(2)_, was chosen. This forward gene selection procedure was continued until fitting was impossible because of the lack of samples. Thusly, a series of K models were built: M_1_ = g_(1)_, M_2_ = g_(1)_ + g_(2)_, …, M_K−1_ = g_(1)_ + g_(2)_ + … + g_(K−1)_, M_K_ = g_(1)_ + g_(2)_ + … + g_(K)_.
4. Akaike information criteria (AICs) for all these models were calculated to avoid overfitting, and the optimal model with the smallest AIC was chosen. The model that is best according to AIC is the one that minimizes prediction error [14, 15].

### 2.5 Construction of the gene prognostic nomogram

The R package “rms” was applied to build the prognostic nomogram based on the expression level of the best genes that were selected by the last step. In the package, the “cph” function was used to construct the COX model. Based on the model, the “nomogram” function was used to generate the prognostic nomogram. The length of the line corresponding to each gene in the prognostic nomogram reflects the contribution of each gene to one patient’s outcome.

### 2.6 Internal and external validation of the gene prognostic nomogram

After the nomogram was constructed, the training set and the validation set were used as the internal validation dataset and the external validation dataset respectively. For these cohorts, we calculated the area under curve (AUC) and the C-index to test the effectiveness of the gene prognostic nomogram in discriminating the outcome of patients. In addition, we generated Kaplan-Meier curves for the high-risk group and low-risk group determined by the cut-off point of the ROC curve. Univariate and multivariate cox regression were performed with the nomogram and molecular subtypes in validation set. The expression of the 6 genes in the nomogram between tumor and normal tissues was compared at the mRNA level in the TIMER database (https://cistrome.shinyapps.io/timer/). cBioportal for Cancer Genomics was explored to investigate the genetic alterations of the prognostic genes in the model. In validation set, log-rank tests (Events were defined as bone metastases.) were performed for the genes in nomogram to see whether these genes were associated with bone metastases in breast cancer.

### 2.7 Statistical analyses

Log-rank tests were performed to preliminarily identify candidate genes for modeling with a P-value less than 0.01 using the R package “survival”. The selection of the best genes for constructing a gene prognostic nomogram was implemented with the R package “rbsurv” [14]. The AUC values were calculated using the R package “timeROC” [16]. These analyses were performed using the R Version 3.5.1(http://www.rproject.org). Univariate and multivariate cox regression were performed using SPSS 25.0 (The alpha level was set as 0.05).

## 3. Results

### 3.1 Selection of microarray datasets for further analyses

There were 935 breast cancer datasets in the GEO database of NCBI. Among them, 2 datasets (GSE5327 [17] and GSE2603 [18]) containing complete information about lung metastasis were selected for further analyses. Their platforms are both GPL96. GSE5327 was considered as the training set and GSE2603 was considered as the validation set. The characteristics of all datasets used in this study are shown in Table 1. The 58 cases of GSE5327 are estrogen receptor (ER) negative, no adjuvant treatment and node negative cancer, without data about progesterone receptor (PR) and HER2 (ERBB2). The 82 cases of GSE2603 include ER, PR and HER2 status and have no data about adjuvant treatment.

**Table 1:** The characteristics of the datasets used in this study.

Dataset	Sample Size	Tissue	Platform
GSE5327	58	Breast Cancer	GPL96
GSE2603	82	Breast Cancer	GPL96

### 3.2 Genes associated with lung metastasis in breast cancer

**Fig 1:**
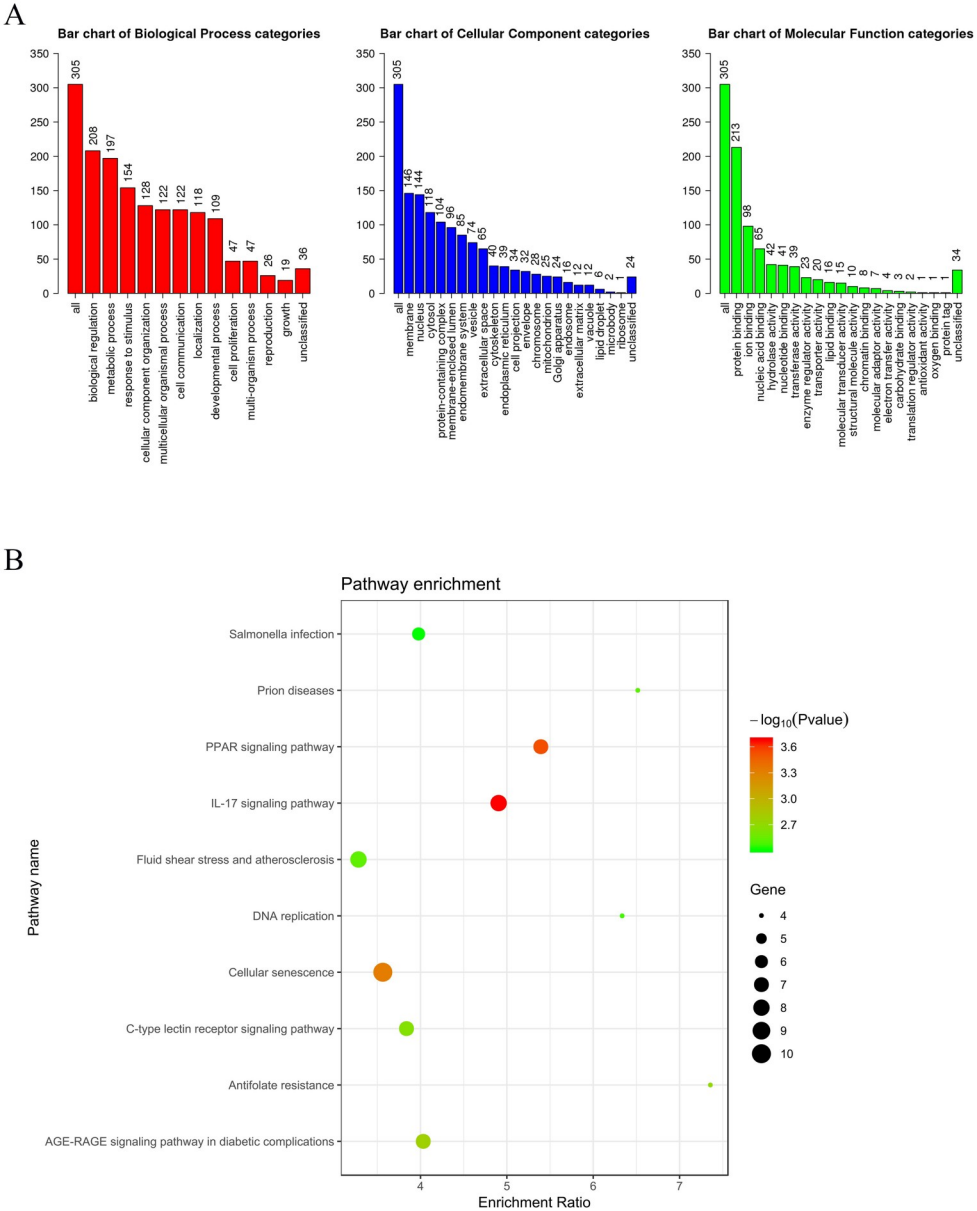
The enrichment results of the genes associated with lung metastasis. (A) Each GO Biological Process, Cellular Component and Molecular Function category is represented by a red, blue and green bar, respectively. The height of the bar represents the number of genes observed in the category. (B) The top 10 enriched KEGG pathway terms of the genes associated with lung metastasis.

We preliminarily identified 319 candidate genes for modeling with a P-value less than 0.01 using the log-rank test (S1 Table). To understand the critical biological significance of the identified genes associated with lung metastasis in breast cancer, enrichment analyses of GO function and KEGG pathways within the identified genes were conducted. The full lists of GO terms are shown in Fig 1A. Of the GO biological process categories, these genes were closely associated with the “biological regulation” and “metabolic process” terms. Of the GO cellular component categories, these genes were closely associated with the “membrane” and “nucleus” terms. Of the GO molecular function categories, these genes were closely associated with the “protein binding” and “ion binding” terms. In addition, the top 10 enriched KEGG pathway terms of the identified genes are listed in Fig 1B.

### 3.3 Best genes for constructing prognostic nomogram

Applying the partial likelihood of the Cox proportional hazard regression model, we next selected the best lung metastasis-associated genes in breast cancer. We used a cross-validation technique considering the large data variability. Forward selection was implemented to build a series of gene models, and the optimal model was then determined using the minimal AIC. Finally, 6 genes (GOLGB1, TMEM158, CXCL8, MCM5, HIF1AN, and TSPAN31) were selected that could optimally predict the lung metastasis risk of breast cancer patients (Table 2).

**Table 2:** The best genes predicting LMFS of breast cancer patients.

Gene Symbol	nloglik	AIC	Selected
GOLGB1	79.62	161.23	*
TMEM158	75.41	154.83	*
CXCL8	70.83	147.66	*
MCM5	69.06	146.12	*
HIF1AN	67.28	144.56	*
TSPAN31	65.32	142.65	*
IFT46	64.89	143.78	
SLC9A3R1	64.89	145.77	
MAPT	64.73	147.46	

### 3.4 The construction of a prognostic nomogram

**Fig 2:**
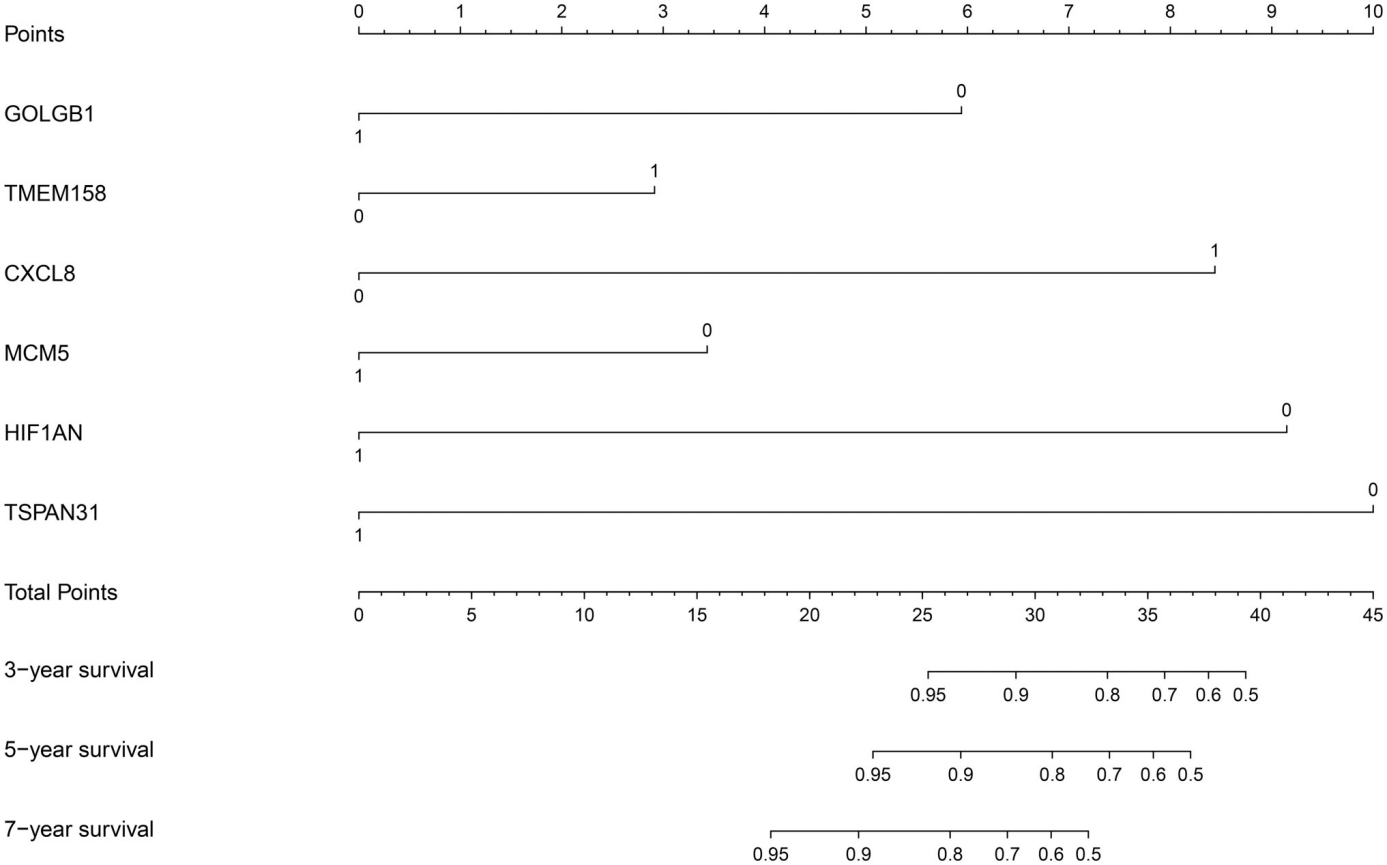
The 6-gene prognostic nomogram based on the expression level of GOLGB1, TMEM158, CXCL8, MCM5, HIF1AN, and TSPAN31. The high and low expression level of each gene were represented by “1” and “0” respectively. “Points” is the score corresponding to the expression level of a single gene. “Total points” is the sum of the “Points” that 6 genes get. The greater “Total points” value means the higher lung metastasis risk of breast cancer patients.

The R package “rms” was applied to construct the prognostic nomogram based on the expression level of the 6 genes (GOLGB1, TMEM158, CXCL8, MCM5, HIF1AN, and TSPAN31). As shown in Fig 2, "1" represents a high expression level of each gene, and "0" represents a low expression level of each gene. “Points” is the score corresponding to the expression level of a single gene. “Total points” is the sum of the “Points” of the 6 genes, which corresponds to the accurate lung metastasis-free survival rate of each sample. A greater “Total points” value indicates a higher lung metastasis risk for breast cancer patients.

### 3.5 Internal and external validation of the prognostic nomogram

**Fig 3:**
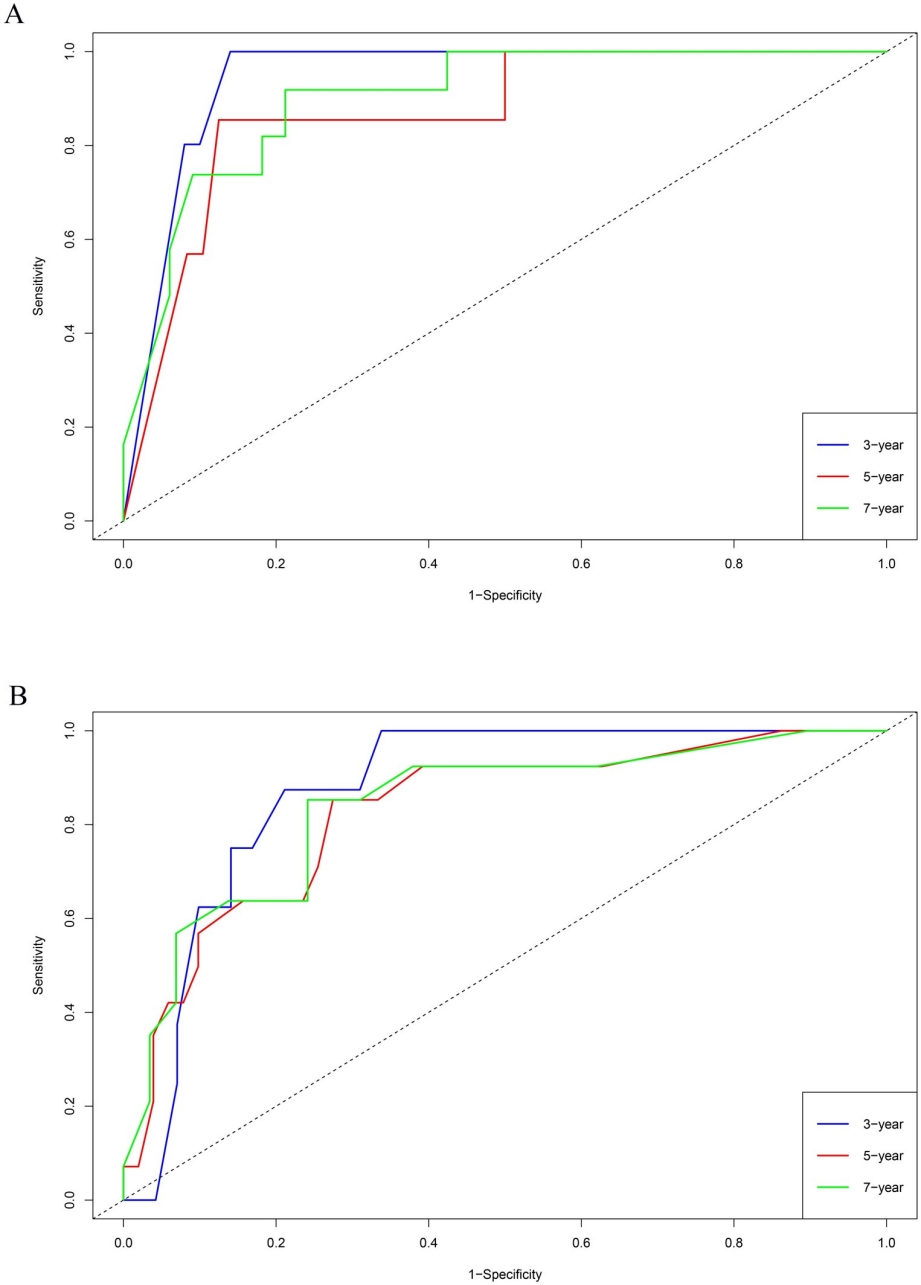
Performance of the 6-gene prognostic nomogram in discriminating lung metastasis risk of breast cancer patients from the GSE5327 and GSE2603 cohorts. (A) For the training set GSE5327, the AUC for 3-, 5- and 7-year were 0.94, 0.87 and 0.90 respectively. (B) For the validation set GSE2603, the AUC for 3-, 5- and 7-year were 0.87, 0.83 and 0.84 respectively.

**Fig 4:**
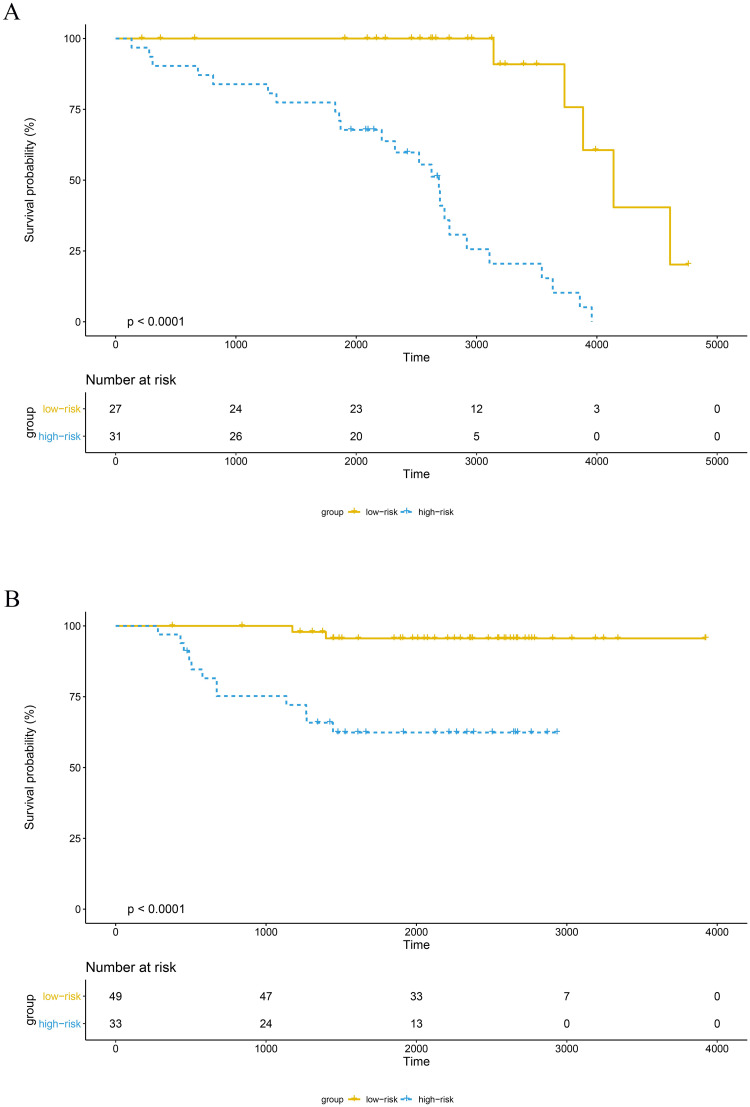
The survival curves of the high-risk and low-risk groups of the GSE5327 and GSE2603 cohorts. (A) In the GSE5327 cohort, the high-risk group exhibited a higher lung metastasis risk than the low-risk group (P<0.0001, cutoff = 18.7). (B) In the GSE2603 cohort, the high-risk group exhibited a poorer prognosis than the low-risk group (P<0.0001, cutoff = 12.0).

**Fig 5:**
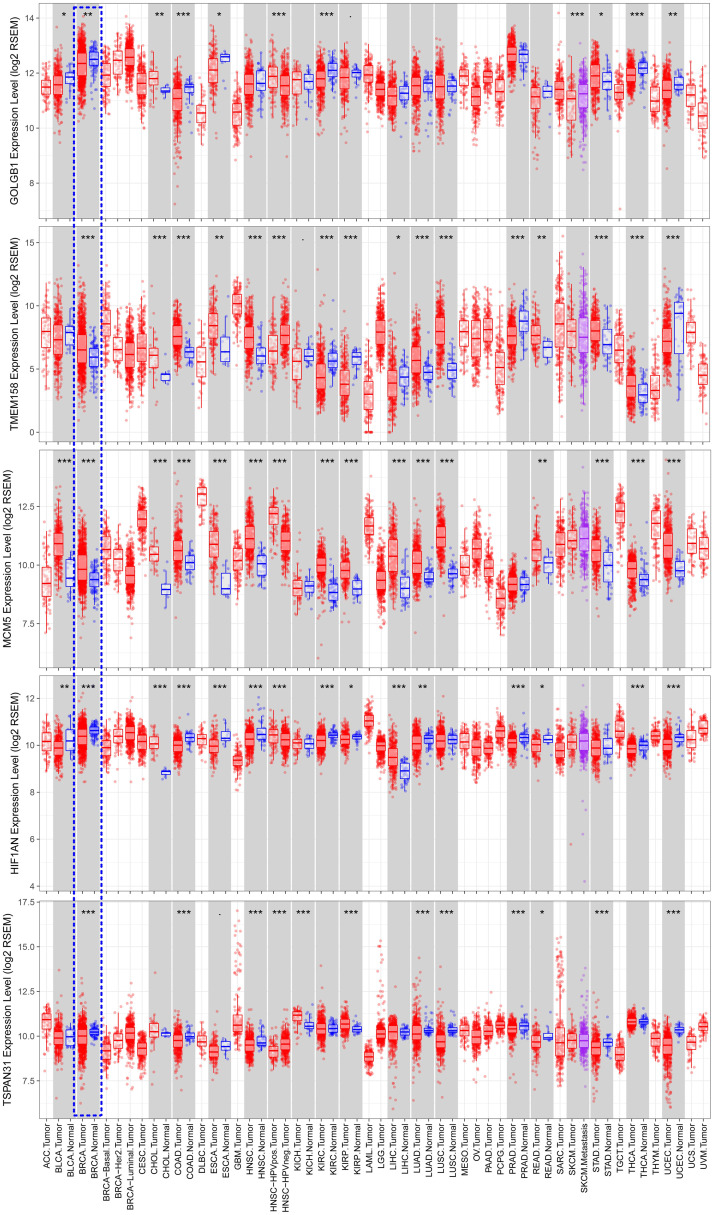
Comparison of expression levels of the 5 prognostic genes between breast cancer and normal breast tissues using data from the TIMER database. *** < 0.001, ** < 0.01, * < 0.05.

**Fig 6:**
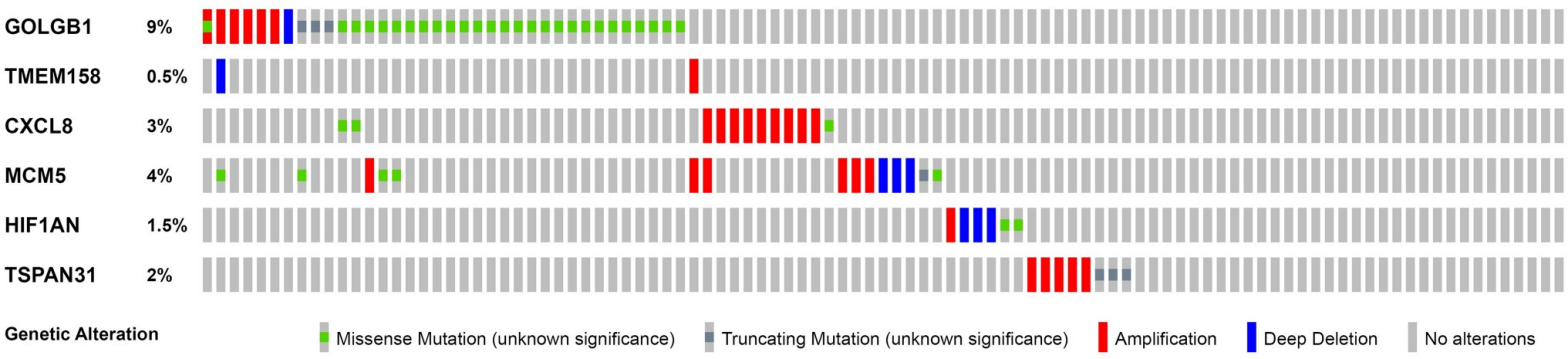
Genetic alterations of the 6 prognostic genes in the nomogram using data from the cBioportal for Cancer Genomics.

**Fig 7:**
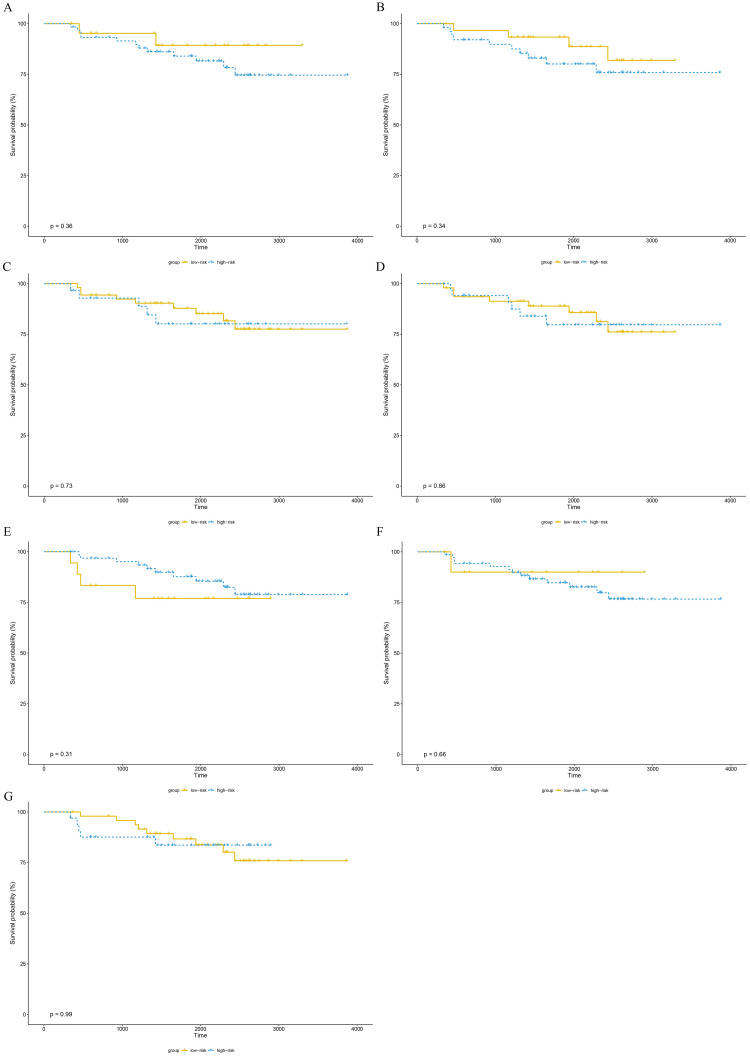
The bone metastasis survival curves of the 6 genes and nomogram in GSE2603 cohorts. (A) GOLGB1. (B) TMEM158. (C) CXCL8. (D) MCM5. (E) HIF1AN. (F) TSPAN31. (G) nomogram. All P-values were greater than 0.05. The cutoff of each gene is its median expression level in the training set. The cutoff of nomogram is consistent with that in Fig 4B.

**Fig 8:**
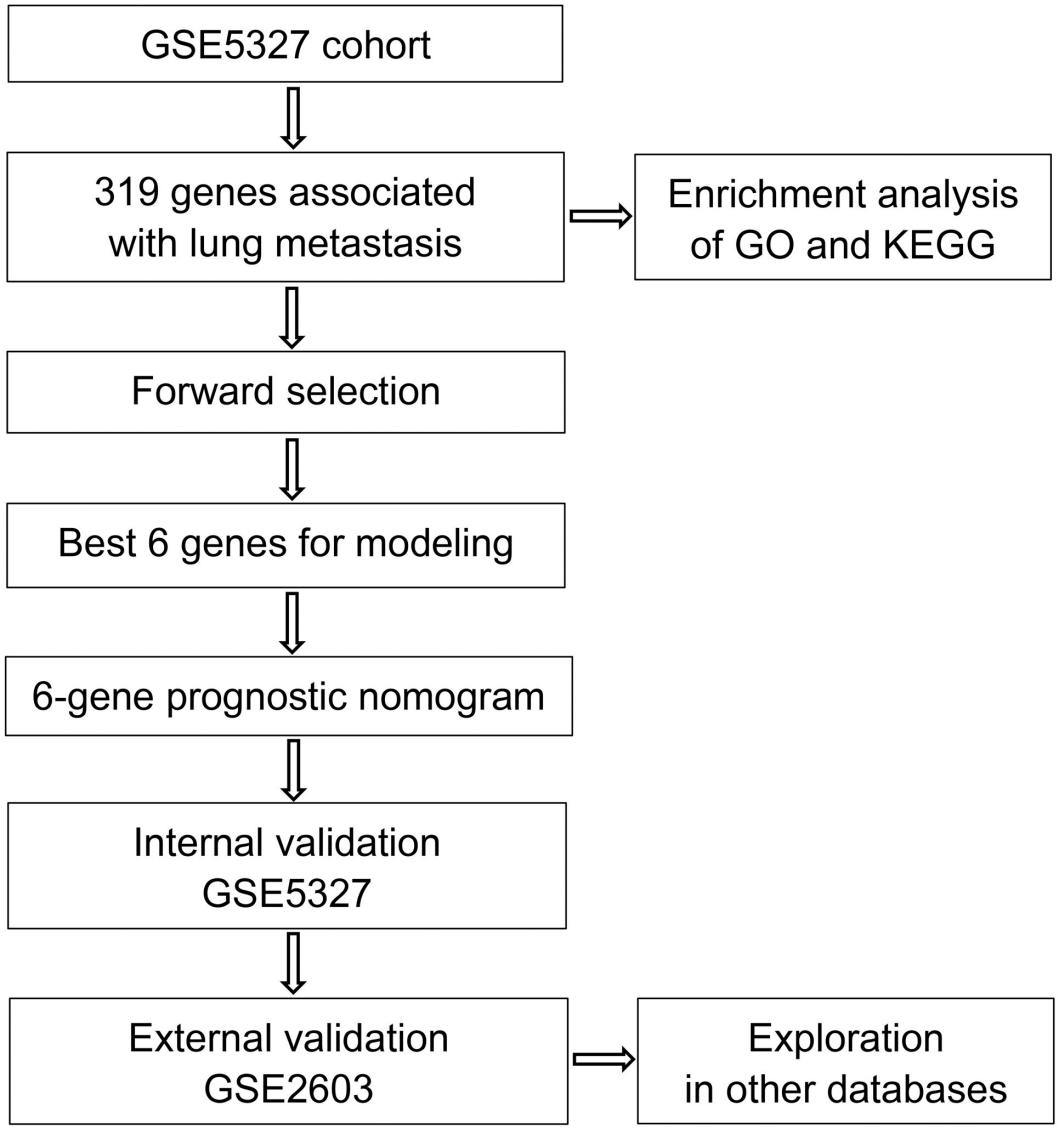
The process of developing the 6-gene prognostic nomogram. First, 319 DEGs associated with lung metastasis in breast cancer patients were identified by univariate survival analysis. Enrichment analysis of GO functions and KEGG pathways was conducted based on these genes. Next, a robust likelihood-based survival modeling approach was applied to identify the best genes for prognosis prediction. Then, the gene prognostic nomogram was constructed based on 6 genes (GOLGB1, TMEM158, CXCL8, MCM5, HIF1AN, and TSPAN31). Finally, the 6-gene prognostic nomogram was validated in the training and validation datasets.

The training set GSE5327 and the validation set GSE2603 were used to evaluate the robustness and effectiveness of the gene prognostic nomogram. The estimated median of LMFS is 3542 days (95% CI: 2821–4264) for GSE5327, not reached for GSE2603. We next generated the time-dependent ROC curve (Fig 3) and calculated the AUC and the C-index for these two datasets. The following values were obtained: for the training set GSE5327, the AUC for 3-, 5- and 7-year were 0.94, 0.87 and 0.90 respectively, and the C-index was 0.862 (P<0.0001); for the validation set GSE2603, the AUC for 3-, 5- and 7-year were 0.87, 0.83 and 0.84 respectively, and the C-index was 0.772 (P<0.0001). These results validated the capability of our gene prognostic nomogram to discriminate the outcome of patients according to the prediction risk. On the other hand, each cohort was divided into a high-risk group and a low-risk group determined by the cut-off point of the ROC curve. It should be noted that the Kaplan-Meier curves showed that lung metastasis was more likely to happen in the high-risk group than in the low-risk group, both in the internal validation set (P<0.0001, Fig 4A) and the external validation set (P<0.0001, Fig 4B). Then, we performed univariate and multivariate cox regression with our nomogram and the molecular subtypes (ER, PR and HER2) in GSE2603. The results showed that both our nomogram and ER status were independent factors for breast cancer lung metastasis (Table 3). Hence, our prognostic nomogram based on 6 genes could effectively predict the lung metastasis risk of patients with breast cancer. What’s more, except for CXCL8 which was not found in the TIMER database, the other five genes were significantly differentially expressed between breast cancer and normal breast tissues (Fig 5). In addition, GOLGB1 possessed the most frequent genetic alterations (9%) in cBioPortal for Cancer Genomics (Fig 6). On the other hand, in the validation set GSE2603, we found by log-rank tests that neither the six genes in the nomogram nor the risk predicted by the nomogram were associated with bone metastasis of breast cancer, preliminarily suggesting that these genes and nomogram were specifically associated with lung metastasis of breast cancer (Fig 7). The whole research process is shown in Fig 8.

**Table 3:** Univariate and multivariate cox regression with molecular subtypes.

Variables	Univariate cox regression			Multivariate cox regression		
	HR	95% CI	P value	HR	95% CI	P value
**ER**	0.110	0.025–0.491	0.004	0.193	0.038–0.972	0.046
**PR**	0.017	0.000–1.236	0.062			
**HER2**	0.508	0.114–2.269	0.375			
**Nomogram**	1.094	1.042–1.149	<0.001	1.061	1.003–1.122	0.039

## 4. Discussion

In this study, we constructed a 6-gene prognostic nomogram that showed its capability to predict the lung metastasis risk for patients with breast cancer. Applying this tool, we could predict which breast cancer patients had a higher risk of lung metastasis and need more attention on their lungs. Within the univariate survival analysis of the breast cancer samples from GSE5327, a total of 319 genes were identified to be associated with the lung metastasis of breast cancer patients. By KEGG analysis, we found that these identified genes were enriched in the signaling pathways such as “PPAR signaling pathway” and “IL-17 signaling pathway”. A previous study showed that the PPAR signaling pathway may be an essential predictor of genes involved in the chemotherapy response for breast cancer patients [19]. In addition, notably, IL-17 family plays an important role in the specific organ metastasis of breast cancer: one reported mouse model manifests that IL-17A leads metastases to the lungs and bones [20, 21]; IL-17E is proposed to be related to lung metastasis formation [22, 23].

After identifying the critical GO function and KEGG pathways, we further selected the best 6 genes to construct the gene prognostic nomogram: GOLGB1, TMEM158, CXCL8, MCM5, HIF1AN, and TSPAN31. GOLGB1 (golgin B1) is reported to be involved in the process of the Golgi affecting tumor progression and metastasis [24]. TMEM158 (transmembrane protein 158) has been proposed to participate in anti-tumor responses [25] and is differentially expressed in triple negative breast cancer [26]. CXCL8 (C-X-C motif chemokine ligand 8) is correlated with clinical breast cancer stage and lymph node metastasis [27]. It has also been indicated that a higher level of CXCL8 promotes the invasive capacity of breast cancer cells [28]. MCM5 (minichromosome maintenance complex component 5) is considered to be a specific target for the gene therapy [29] and a biomarker associated with the relapse-free survival of breast cancer patients [30]. HIF1AN (hypoxia inducible factor 1 subunit alpha inhibitor) was found to be correlated with the absence of lymph node metastasis [31]. In addition, there is one report concerning TSPAN31 (tetraspanin 31) and lung metastasis happened in osteosarcoma [32]. None of these six genes had previously been reported to be associated with lung metastasis in breast cancer. Meanwhile, we found in the TIMER database that the aberrant expression of five genes (GOLGB1, TMEM158, MCM5, HIF1AN, and TSPAN31) occurred in a variety of tumors. Their biological roles in the lung metastasis of breast tumor cells would be of great interest in further studies.

The 6-gene prognostic nomogram was validated in both the training set and validation set. The AUC and the C-index revealed the nomogram’s effectiveness in discriminating the outcome of breast cancer patients; The Kaplan–Meier curve showed that the high-risk group had a higher likelihood of lung metastasis. The multivariate analysis showed that both our nomogram and ER status were independent factors for breast cancer lung metastasis. All of these results demonstrate that this prognostic nomogram based on 6 genes has the capability of predicting the lung metastasis risk of breast cancer patients. Meanwhile, the bone metastasis survival curves of the 6 genes and nomogram in validation set preliminarily suggested that these genes and nomogram were specifically associated with lung metastasis of breast cancer, but further research is needed to confirm this conclusion.

Compared with overall survival, metastasis-free survival can better reflect the clinical benefits and prognosis of non-metastatic patients because metastasis is the most important factor that threatens the life of breast cancer patients and hinders the treatment of breast cancer. However, there are differences in the tendency of breast cancer to metastasize to different organs. In this study, we focused on one of the host organs, lung, and built a prognostic nomogram with 6 genes that could effectively predict the lung metastasis risk for breast cancer patients. In addition, the method used to construct the model is flexible and easy. The result is presented as the relative risk combined with the absolute lung metastasis-free survival rate, which is more illustrative and intuitive. Based on the relative risk ratio, the specific lung metastasis-free survival rate of an individual can be queried according to the expression level of the 6 genes, and patients predicted to be at high risk will very likely need more attention and care to their lungs. The prognostic nomogram can be conveniently constructed in the R environment and can serve as a robust tool in model prediction. It is essential to make real-time quantitative PCR (QPCR) assays more popular in the clinic. The expression level of genes could be obtained using QPCR, so our gene nomogram can be conveniently implemented in routine clinical settings. The process of constructing this model would provide a good reference for the prediction of tumor metastasis to other specific organs, such as the bone and brain.

However, there were several limitations in this study. First, due to the limited number of datasets on breast cancer lung metastasis, the training set did not contain complete information about molecular subtypes, and the validation set did not contain information about adjuvant treatment. We were unable to construct a model that adjusted for these prognostic factors. Second, other important outcomes, such as overall survival, were not recorded in these two datasets. It would be meaningful to investigate the relationship between our nomogram and other outcomes. In our future studies, we will collect more clinical breast cancer tissues with concrete metastasis information from our own hospitals to establish predictive tools for other metastasis sites. Meanwhile, other well-known clinical prognostic factors (such as molecular subtypes and adjuvant treatment) and important outcomes (such as overall survival) that could not be obtained from the database, should be the focus of our next study. With a more comprehensive collection of patient clinical information, we will try to build more accurate and efficient models for predicting specific organ metastasis.

## Supporting information

S1 Table (XLSX)
